# High viral suppression and detection of dolutegravir-resistance associated mutations in treatment-experienced Tanzanian adults living with HIV-1 in Dar es Salaam

**DOI:** 10.1038/s41598-023-47795-1

**Published:** 2023-11-22

**Authors:** George M. Bwire, Beatrice Godwin Aiko, Idda H. Mosha, Mary S. Kilapilo, Alli Mangara, Patrick Kazonda, Janeth P. Swai, Omary Swalehe, Michael R. Jordan, Jurgen Vercauteren, David Sando, David Temba, Amani Shao, Wilhellmuss Mauka, Catherine Decouttere, Nico Vandaele, Raphael Z. Sangeda, Japhet Killewo, Anne-Mieke Vandamme

**Affiliations:** 1 Laboratory of Clinical and Epidemiological Virology (Rega Institute), Department of Microbiology, Immunology and Transplantation, Rega Institute for Medical Research Clinical and Epidemiological Virology, Institute for the Future, KU Leuven, Rega-Herestraat 49-Bus 1040, 3000 Leuven, Belgium; 2 Department of Pharmaceutical Microbiology, School of Pharmacy, Muhimbili University of Health and Allied Sciences, Dar es Salaam, 65013 Tanzania; 3 Department of Pharmaceutics and Pharmacy Practice, School of Pharmacy, Muhimbili University of Health and Allied Sciences, Dar es Salaam, 65013 Tanzania; 4 Faculty of Economics and Business, Access to Medicine Research Center, KU Leuven, 3000 Leuven, Belgium; 5 Department of Behavioural Sciences, School of Public Health and Social Sciences, Muhimbili University of Health and Allied Sciences, Dar es Salaam, 65015 Tanzania; 6 Department of Epidemiology and Biostatistics, School of Public Health and Social Sciences, Dar es Salaam Urban Cohort Study, Dar es Salaam, 65013 Tanzania; 7 Department of Business Studies, School of Business, Mzumbe University, Dar es Salaam, 20266 Tanzania; 8 Tufts University School of Medicine, Boston, MA USA; 9 Managament and Development for Health, Mwai Kibaki Road, Dar es Salaam, Tanzania; 10 Department of Epidemiology and Biostatistics, School of Public Health and Social Sciences, Muhimbili University of Health and Allied Sciences, Dar es Salaam, 65015 Tanzania; 11 Center for Global Health and Tropical Medicine, Instituto de Higiene e Medicina Tropical, Universidade Nova de Lisboa, Lisbon, Portugal

**Keywords:** HIV infections, Epidemiology

## Abstract

To curb HIV infection rate in Tanzania, antiretroviral therapy (ART) has been scaled up since 2006, and in 2019, the country shifted to regimen including dolutegravir as a default first line. We assessed the success of ART and the contribution of HIV drug resistance (HIVDR) to unsuppressed viral loads. Between February and May 2023 a cross-sectional survey with random sampling was conducted in the six clinics in an urban cohort in Dar es Salaam. Patients with unsuppresed viral loads (local criteria viral load (VL) ≥ 1000 copies/mL) were tested for HIVDR mutations using the WHO adapted protocol for plasma samples. Mutations were interpreted using the Stanford HIVDR database. In total 600 individuals participated in this survey, the majority were female (76.83%), mean age ($\pm$ standard deviation) was 44.0 ($\pm$ 11.6) years. The median duration on ART (interquartile range) was 6.5 (3.9–10.2) years. Approximately 99% were receiving tenofovir + lamivudine + dolutegravir as a fixed dose combination. VL testing was successful in 99.67% (598/600) of survey patients and only 33 had VL ≥ 1000 copies/mL, resulting in a viral suppression level of 94.48% (565/598, 95% CI 92.34–96.17%). For 23 samples, protease and reverse transcriptase (RT) genotyping were successful, with 13 sequences containing RT inhibitor surveillance drug resistance mutations (SDRMs) (56.5%). No SDRM against protease inhibitors were detected. Thirty samples were successfully genotyped for integrase with 3 sequences (10.08%) containing integrase strand transfer inhibitor (INSTI) SDRMs. In samples successfully genotyped in the three genetic regions, 68.18% (16/22) had a genotypic susceptibility score (GSS) ≥ 2.5 for the concurrent regimen, implying factors beyond drug resistance caused the unsuppressed viral load. For five patients, GSS indicated that HIVDR may have caused the unsuppressed viral load. All three patients with INSTI resistance mutations were highly resistant to dolutegravir and accumulated nucleoside and non-nucleoside RT inhibitor HIVDR mutations. Although in this cohort the last 95 UNAIDS target was almost achieved, HIVDR mutations, including INSTIs resistance mutations were detected in HIV-positive individuals taking ART for at least one year. We recommend the design and implementation of high-impact interventions to prevent the increase of HIVDR, failure of dolutegravir and address the non-resistance factors in the study area.

## Introduction

As of 2021, 38.4 million people were infected with the human immunodeficiency virus (HIV) globally^1^, 1.7 million of them living in Tanzania. The 2021 national HIV survey ranked Dar es Salaam region among the top three with the highest HIV prevalence. Mbeya region had the highest prevalence (14%), followed by Iringa region (13%) and Dar es Salaam region (11%)^2^. To curb the HIV epidemic, UNAIDS put forward the 95–95–95 targets for 2025: 95% of all people living with HIV are diagnosed, 95% of diagnosed are treated, with 95% of them achieving viral load suppression^3^. Worldwide, 75% of all people living with HIV have currently access to antiretroviral therapy (ART) and more than 1.3 million in Tanzania (76% of diagnosed) are receiving ART^4^.

In 2006, Tanzania scaled-up the use of ART for treatment of HIV-1 infection^5^. Criteria to start and change therapy are currently following the country-wide policy to test and treat with a default first line therapy and move to a default second line therapy when viral load rises above 1000 copies/ml for two consecutive tests, amounting to therapy failure in the Tanzanian context^2^. The country adopted a triple drug first-line ART regimen, consisting of two nucleoside reverse transcriptase inhibitors (NRTIs) combined with either a non-nucleoside reverse transcriptase inhibitor (NNRTI) or a protease inhibitor. The initial default first-line ART regimen included nevirapine based regimen (tenofovir, lamivudine and nevirapine)^6^. Later, nevirapine was replaced by efavirenz, and in 2019, the country shifted to using an integrase inhibitor (dolutegravir)^7^. This shift was for all patients, whether they were starting treatment or whether they were already on a first line regimen. Shifting to integrase inhibitors was influenced by the reported high-level drug resistance to, and poor virologic outcomes associated with the use of NNRTIs^8^.

Dolutegravir (DTG), an integrase strand transfer inhibitor (INSTI), has proven highly effective in suppressing HIV replication when used in combination ART^9^. DTG-based regimen, specifically the combination of tenofovir, lamivudine and DTG (TLD) is now the preferred first-line regimen for adults living with HIV in Tanzania^7^. This regimen has demonstrated high effectiveness in suppressing viral load, is cost-effective, well-tolerated by patients^10–12^ and has high genetic barrier to resistance^13^. TLD is described to be an important milestone for treated patients in suppressing the viral replication^11,12^. However, the transition to DTG is now facing challenges of emerging HIV resistance to INSTIs^14,15^ and circulating HIV integrase genotypes linked to drug resistance^16^. Resistance to DTG has emerged in various settings^13^. Common dolutegravir resistance mutations, such as R263K, G118R, H51Y, and N155H, diminish drug effectiveness by reducing its binding affinity to the integrase enzyme^17^.

The national HIV survey conducted in 2021 revealed that approximately 17% (221,000) of those on ART did not achieve VL suppression defined as having two consecutive viral loads ≥ 1000 copies/mL^4^. This unsuppressed viral load can be attributed to not correctly taking the therapy (e.g., due to adherence or counselling problems), or to HIV drug resistance (HIVDR) which could be pre-existing or newly emerging drug resistance mutations^14,18,19^.

To protect the current and future activity of regimens with INSTI inhibitors, the scale of the problem needs to be assessed. This calls for regular monitoring and surveillance of drug resistance^20^, in addition to the viral load testing for routine therapy follow up, in order to provide timely data on the emerging and timing of dolutegravir resistance^21^. This monitoring and surveillance may not only help to inform treatment algorithms for patients whose dolutegravir-based ART may fail, but it can also provide crucial information to inform future HIV treatment guidelines^22^ and prevent possible emergence of dolutegravir resistance. Furthermore, monitoring of emerging HIVDR could help protect the long-term efficacy of available treatment regimens^23^.

In a conceptual systems map, Kiekens et al.^24^ indicated three main loops contributing to HIVDR in sub-Saharan Africa. One of these is that overreliance on new drugs with a high genetic barrier to resistance contributes to an increase in HIVDR. To avoid this to come true, a systems approach is needed and quantitative data need to be collected. HIVDR surveillance data and its association with other factors will contribute to assess the problem of HIVDR at a systems level and uncover potential leverage points that could be addressed with intervention^25^. In this study we used the Dar es Salaam urban cohort of patients from the Ilala district to determine the virologic suppression rate and the prevalence of HIVDR as well as the circulating resistance patterns. We also determined the factors associated with unsuppressed viral loads. With this information, a baseline for intervention studies at the study site will be available.

## Materials and methods

### Study design and patient

A cross-sectional survey was conducted between February and May 2023 in the Dar es Salaam Urban Cohort Study (DUCS) platform of the Ilala district in Dar es Salaam^26^. DUCS area (Ukonga and Gongolamboto administrative wards) has a total of six HIV care and treatment clinics (CTCs); three of which are public owned facilities, two are military owned while one is privately owned (faith-based). All adults (18 years and above) living with HIV and resident in the DUCS platform area and receiving antiretroviral therapy in CTCs found in the DUCS area were eligible to participate in the study. Patients who had received ART for less than one year were excluded from the study.

### Sample size and sampling technique

The sample size (n) was computed using a cross sectional formulae for a finite patient; *n* = *N(z*^*2*^*) p(1-p)*/*d*^*2*^*(N-1)* + *(z*^*2*^*) p(1-p)*^27^ based on the assumption of an estimated prevalence (p) of 0.68%^15^ for detecting any HIVDR in adult Tanzanian patient taking ART. Using type I error of 5% (z = 1.96) and a margin of error (d) of ± 0.55%, a minimum sample size of 565 patients was required. With an oversampling of 20%, to allow for non-responders, drop outs and failures in testing, 720 from the 1651 eligible patients were selected using a simple random sampling (using SPSS version 29, Chicago Inc., USA)^28^. Furthermore, a proportional sampling method was used to obtain a representative sample from all six HIV CTCs found in the DUCS platform area.

### Patient recruitment procedures

Phone contacts were extracted from patient files, with up to three call attempts. Patients visiting the clinic were informed about the study, provided informed consent, completed a questionnaire on electronic tablets, and had venous blood drawn. Regardless of consent status, patients received reimbursement for transport costs and a one-kilogram sugar incentive. This process ensured an organized and incentive-driven approach to patient recruitment and study participation.

### Data collection process

The study questionnaire was designed following a comprehensive literature review^12,24,25,29,30^. It consisted of questions about patient social demographic information such as; age, sex, employment status, place of residence and marital status, questions designed to reflect on factors associated with HIVDR such as HIV status disclosure and health insurance. Patient clinical information such as regimen used, treatment line and duration on ART were collected from the record files of the patients (Table 1).

**Table 1:** Patients’ socio-demographic, clinical characteristics, and their association with unsuppressed viral loads.

Variable	Category	Overall (N = 600), n (%),	Viral load count (copies/mL), N = 598		Association with unsuppresed viral loads (VL ≥ 1000 copies/ml)					
					Univariate			Multivariate		
			< 1000, n (%)	$\ge$ 1000, n (%)	cOR	95% CI	P-value	aOR	95% CI	P-value
Sex	Male	139 (23.16)	129 (22.83)	10 (30.30)	0.79	0.36–1.75	0.557	0.54	0.18–1.62	0.270
	Female	461 (76.83)	436 (77.16)	23 (69.69)	Reference					
Age (years)	Mean (SD), 44.0 (11.6) years									
	18–24	17 (2.83)	15 (2.65)	2 (6.06)	0.48	0.07–3.26	0.453	0.09	0.00–3.14	0.189
	25–34	87 (14.50)	83 (14.69)	4 (12.12)	0.47	0.09–2.58	0.385	0.08	0.00–2.45	0.150
	35–44	203 (34.16)	191 (33.80)	13 (39.39)	0.30	0.05–1.78	0.185	0.03	0.00–1.184	0.062
	45–54	177 (29.50)	169 (29.91)	7 (21.21)	0.36	0.06–2.11	0.256	0.04	0.00–1.45	0.079
	≥ 55	114 (19.00)	107 (18.93)	7 (21.21)	Reference					
Highest education level	Primary	454 (75.66)	425 (75.22)	29 (87.87)	0.80	0.26–2.42	0.691	0.37	0.07–1.94	0.238
	Secondary	97 (16.16)	92 (16.28)	4 (12.12)	–	–	–	–	–	–
	College/ university	19 (3.16)	18 (3.18)	0 (0.00)	–	–	–	–	–	–
	Not attended any formal education	30 (5.00)	30 (5.30)	0 (0.00)	Reference					
Employment status	Employed in public sector	80 (13.33)	76 (13.45)	4 (12.12)	0.83	0.26–2.66	0.757	0.53	0.13–2.14	0.377
	Employed in private sector	347 (57.83)	330 (58.40)	16 (48.48)	–	–	–	–	–	–
	Self-employed	5 (0.83)	5 (0.88)	0 (0.00)	3.1	0.65–14.73	0.155	3.40	0.47–30.65	0.210
	Farmer/peasant	39 (6.50)	35 (6.19)	4 (12.12)	1.40	0.40–4.92	0.605	1.56	0.33–7.36	0.573
	House wife/husband	129 (21.50)	119 (21.16)	9 (27.27)	Reference					
Microfinance membership	Yes	116 (19.33)	112 (19.82)	3 (9.09)	2.34	0.68–7.98	0.176	2.23	0.56–8.90	0.257
	No	483 (80.66)	453 (80.17)	30 (90.90)	Reference					
Marital status	Married	268 (44.66)	252(44.60)	16 (48.48)	0.73	0.25–2.11	0.557	1.09	0.28–4.35	0.899
	Not married	107 (17.83)	101 (17.87)	5 (15.15)	0.62	0.17–2.29	0.478	1.34	0.28–6.49	0.719
	Separated	60 (10.00)	56 (9.91)	3 (9.09)	0.74	0.26–2.16	0.587	0.78	0.17–3.63	0.755
	Widow/widower	90 (15.00)	85 (15.04)	5 (15.15)	0.78	0.21–2.89	0.712	1.15	0.23–5.91	0.866
	Divorced	64 (10.66)	61 (10.79)	3 (9.09)	1.56	0.16–14.88	0.698	3.61	0.12–106.43	0.458
	Not in any relationship	11 (1.83)	10 (1.76)	1 (3.03)	Reference					
Having children	Yes	553 (92.16)	520 (92.03)	31 (93.93)	0.81	0.18–3.65	0.780	0.14	0.00–2.51	0.183
	No	47 (7.83)	45(7.96)	2 (6.06)	Reference					
Type of CTC facility	Public-owned	147 (24.50)	139 (24.60)	7 (21.21)	1.22	0.48–3.12	0.678	0.94	0.30–2.92	0.92
	Military-operated	296 (49.33)	280 ( 49.55)	16 (48.48)	1.45	0.52–4.05	0.477	1.4	0.38–5.22	0.62
	Private-owned	157 (26.16)	146 (25.84)	10 (30.30)	Reference					
Average waiting time at the clinic (min)	Median (IQR), 20.0 (10.0–30.0) min		116 (19.33)	112 (19.82)	3 (9.09)					
	< 30	334 (55.66)	316 (55.92)	17 (51.51)	1.10	0.50–2.40	0.822	1.53	0.54–4.33	0.418
	31–59	213 (35.50)	200 (35.39)	12 (36.36)	2.02	0.61–6.78	0.250	2.67	0.62–11.49	0.186
	≥ 60	53 (8.83)	49 (8.67)	4 (12.12)	Reference					
Time since first HIV-positive test (years)	Median (IQR), 6.8 (4–10.5) years									
	< 2	90 (15.05)	83 (14.69)	7 (21.21)	0.27	**0.07**–**0.97**	**0.045**	0.13	**0.02**–**0.69**	**0.017**
	2–5	172 (28.60)	167 (29.55)	4 (12.12)	1.14	0.43–3.03	0.788	6.63	0.11–371.2	0.357
	6–10	194 (32.27)	177 (31.32)	16 (48.48)	0.53	0.17–1.71	0.289	1.58	0.06–40.37	0.782
	$>$ 10	144 (24.08)	138 (24.42)	6 (18.18)	Reference					
Time since start of HIV treatment (years)	Median (IQR), 6.5 (3.9–10.2) years									
	< 2	45 (7.50)	43 (7.61)	2 (6.06)	0.86	0.17–4.27	0.852	3.18	0.40–25.30	0.273
	2–5	225 (37.50)	214 (37.87)	10 (30.30)	1.83	0.39–8.76	0.445	0.27	0.00–19.05	0.546
	6–10	199 (33.16)	182 (32.21)	16 (48.48)	0.80	0.14–4.52	0.802	0.46	0.01–18.59	0.684
	$>$ 10	131 (21.83)	126 (22.30)	5.(15.16 )	Reference					
ART regimen	TLD	596 (99.33)	561 (99.29)	32 (96.96)	3.77	0.33–42.71	0.285	1.4	0.05–35.21	0.837
	Other regimen*	4 (0.06)	4 (0.70)	1 (3.03)	Reference					
Self-reported chronic disease apart from HIV	Yes	62 (10.33)	56 (9.91)	6 (18.18)	0.34	**0.12**–**0.93**	**0.036**	0.18	**0.05**–**0.73**	**0.016**
	No	538 (89.66)	509 (90.08)	27 (81.81	Reference					
Health insurance	Yes	81 (13.50)	78 (13.80)	3 (9.09)	1.52	0.43–5.26	0.512	1.38	0.31–6.24	0.676
	No	519 (86.50)	487 (86.19)	30 (90.90)	Reference					
HIV disclosure status	Yes	516 (86.00)	486 (86.01)	29 (87.87)	0.79	0.26–2.39	0.681	1.08	0.28–4.15	0.909
	No	84 (14.00)	79 (13.98)	4 (12.12)	Reference					
Viral load count (copies/mL)	< 1000	< 50	502 (83.95)							
		50–999	63 (10.54)							
	≥ 1000		33 (5.52)							

Significant factors were bolded.

*ART* antiretroviral therapy, *TLD* tenofovir + lamivudine + dolutegravir, *cOR* crude odds ratio, *aOR* adjusted odds ratio, *SD* standard deviation, *IQR* interquartile range at 25% and 75%, *CTC* care and treatment clinic.

–: Not reported due to wide confidence interval.

*Other regimen: Tenofovir (TDF) + lamivudine (3TC) + efavirenz (EFV); TD + emtricitabine (FTC) + efavirenz (EFV); TDF + (3TC or FTC) + dolutegravir (DTG); abacavir (ABC) + 3TC + (EFV or DTG); zidovudine (AZT) + 3TC + (EFV or DTG); AZT + 3TC + nevirapine (NVP); TDF + (FTC or 3TC) + atazanavir boosted by ritonavir (ATV/r).

Failed viral suppression: viral load $\ge$ 1000 copies/mL.

### Blood sample handling, processing and storage

Blood sample collection, transportation and storage were treated according to the Temeke Regional Referral Hospital- Specialized Laboratory protocol^31^. Eight ml of venous blood was drawn using two EDTA tubes each with 4 ml. Whole blood was separated into plasma within 6 h of collection, before separation the whole blood was kept at 2–8 °C. Centrifugation was performed for 10 min at 1000–2000×*g*, then supernatant (plasma) was kept at − 20 °C with a maximum of 3 times of freeze–thaw cycles.

### Viral load testing and HIV drug resistance analysis

Viral load testing was performed using Roche cobas® 6800/8800 systems^32^ (software version 1.3, publication 4.2) with a lower limit of detection of 15 copies/mL. Given that we followed the current Tanzania national HIV treatment guidelines^2^, only samples with viral load count ≥ 1000 copies/mL were subjected to analysis of HIVDR mutations. We used Management and Development for Health (MDH) WHO adapted protocol (Unpublished) to perform HIVDR testing using the 3500xL genetic analyser (Applied Biosystems), a capillary sequencer using Sanger-style sequencing. Analysis of mutations were performed using Stanford University HIVDR database version 9.4. Moreover, calibrated patient resistance tool (CPR version 8.1) was used to determine proportion of mutation suggestive of transmitted HIVDR resistance^33^. Before importing the generated sequences (fasta format) to Stanford University HIVDR database, the raw sequence (abi format) was assembled, aligned and edited using a RECall (v2.32)- web based sequence analysis then saved as fasta file^34^. The sequenced codons were 6–99, 1–251, and 1–288, for protease, reverse transcriptase and integrase proteins, respectively. We used Stanford HIVDR^33^ and COMET^35^ to assign the HIV sub-types, and Rega HIV sub-typing tool 3.46 database^36^ was consulted to resolve any disagreements between Stanford and COMET. The VL testing and the genotyping data were linked to the questionnaire information in the DUCS database for analysis.

### Statistical analysis

Descriptive statistics of the socio-demographic characteristics were summarized using frequencies (n) and percentages (%). In accordance with the Tanzania national HIV treatment guideline^7^, which was adapted from the WHO^37^ guidelines, patients were considered to have a suppressed viral load when the count was < 1000 copies/mL, while ≥ 1000 copies/mL was considered unsuppressed viral load. In addition, in some analysis, viral load counts were stratified into three categories: < 50 copies/mL, 50–999 copies/mL, and ≥ 1000 copies/mL. The viral suppression was calculated by dividing the number of patients with viral load count below 1,000 copies/mL over successfully viral load tested patients. In this study we estimated the prevalence of HIVDR as the proportions of patients’ samples which harboured surveillance drug resistance mutations to those patients’ with successfully genotyped sequences.

We determined the 95% CI for viral suppression and proportion of patients with any major HIVDR mutations using the binomial approximation^38^. We used mean and standard deviations or median and interquartile range (IQR) to estimate the measure of central tendencies for symmetrical and asymmetrical continuous data, respectively. Factors associated with unsuppressed viral loads (1000 copies/mL and above) were determine using binary logistic regression model. Crude and adjusted odds ratios (cOR and aOR) were the effect measures for univariate and multivariate for regression analysis, respectively. All variables in univariate analysis were used in the multivariate analysis. We used the confidence interval of 95% (95%CI), and the p-value $<$ 0.05 was considered statistically significant.

Statistics were done using SPSS software (SPSS version 29, Chicago Inc., USA) and Excel spreadsheet 2022 (Microsoft Corporation, Redmond, WA). The Genotypic Susceptibility Score (GSS) for the combination antiretroviral therapy (regimen) was determined by adding the score of each drug used in the regimen, using the Stanford HIVDB scoring system^39^. The interpretations of these tests were categorized as 'susceptible', 'possible resistance', and 'resistance', which were assigned scores of 1, 0.5, and 0, respectively^40^. Possible resistance included potential low-level resistance, low-level resistance, and intermediate resistance. Patient was considered susceptible to the regimen if the GSS was $\ge$ 2.

### Ethics declarations

The study was approved by Muhimbili University of Health and Allied Sciences (MUHAS)-Ethical Review Board (Ref No. MUHAS/REC/2020/243) and the National Committee on Medical Research Ethics (Ref No. NIMR/HQ/R.8c/Vol.1/1870). Permissions to access the CTCs were requested from the President's Office—Regional Administration and Local Government and CTC managers, Dar es Salaam Regional Medical Officer, Ilala District Medical Officer and CTCs managers. This study complied with the Declaration of Helsinki for medical research involving human subjects.

## Results

### Overview of recruitment and genotyped samples

**Figure 1:**
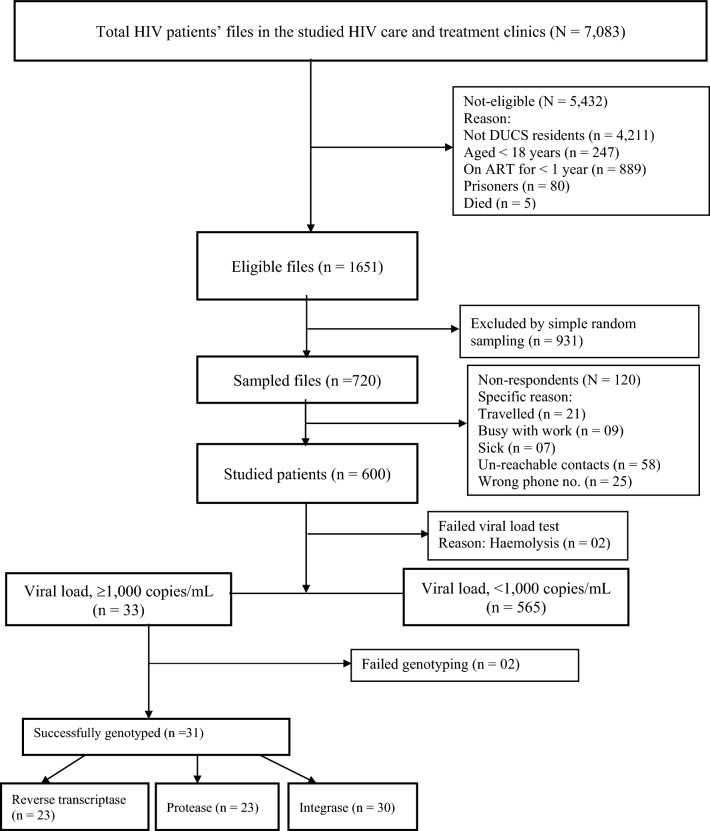
Patients’ recruitment flow chart and testing success rates for the HIVDR survey conducted in Dar es Salaam Urban Cohort Study (DUCS) for patients receiving antiretroviral therapy (ART).

Between February and May 2023, 7083 treated patients were screened from the HIV care and treatment clinics in the DUCS platform for eligibility criteria and 1651 patients were found eligible for inclusion (Fig. 1). With regard to the exclusion criteria, the most common reasons for exclusion were (i) not living in the study area (59.45%); (ii) being on ART for less than one year (12.55%); and, younger than 18 years old (3.48%). None who attended their appointment refused to give informed consent. Viral loads were determined in 600 patients, the success rate was 598 samples (99.67%), with 5.52% (n = 33) having unsuppressed viral load. Genetic sequencing was successful in the protease and reverse transcriptase regions for 23 patients’ samples (69.70%) and in the integrase region for 30 patients’ samples (90.90%).

### Characteristics of the surveyed patients

For the survey patients, we collected socio-demographic and other characteristics potentially associated with unsuppressed viral load, summarized in Table 1. The majority was female, the mean age was 44 years, and the education level was mainly primary education (Table 1). About half of patients were attending the military-operated CTCs, most patients spent less than 30 min waiting during clinic visits, and the median time since first HIV diagnosis was 6.8 years. Approximately, 99% of survey patients were taking TLD regimen.

According to the local criteria of therapy success (VL < 1000 copies/mL) 94.48% (565/598, 95% CI 92.34–96.17%) were successfully treated, which comes close to 95%, the goal of UNAIDS for 2025. Those with a viral load count < 50 copies/mL accounted for 83.95% (502/598), those with 50–999 copies/mL made up 10.54% (63/598), while those with ≥ 1000 copies/mL constituted 5.52% (33/598).

Both univariate and multivariate analysis found an association between unsuppressed viral loads and time since first HIV-positive test, patients diagnosed since less than 2 years had lower odds of having unsuppressed viral load (aOR 0.13, 95% CI 0.02–0.69, p = 0.017) as compared to those with at least 10 years since diagnosis. However, patients with self-reported other underlying chronic disease conditions also had lower odds of unsuppressed viral load (aOR 0.18, 95% CI 0.05–0.73, p = 0.016) as compared to their counterparts (Table 1).

### Characteristics and treatment history of patients with viral load $\ge$ 1000 copies/mL

The mean age (± standard deviation) of participants was 42.97 (± 13.16) years, while the mean duration on ART was 6.79 (± 3.67) years (Table 2). Moreover, mean (± standard deviation) time since the start of dolutegravir-based regimen was 32.6 (± 12.95) months. Out of 33 patients, 32 (96.97%) had been initiated on a DTG-based regimen either as first therapy or switched from another first line, all of which because of a national policy change. Many of these patients also had previous treatment changes, and two patients were switched away from DTG. The reason for previous changes is not known, however, given that all were switched from a first line suggests the previous changes might also have been policy changes or might have been related to side effects.

**Table 2:** Clinical and treatment history for patients with viral load $\ge$ 1000 copies/mL (N = 33).

Participant ID	Age	Sex	Duration on ART years	Previous used regimen*	Duration on dolutegravir based regimen (months)	Reason for change to last regimen
CR-014	45	Female	10	1.TDF + FTC + EFV 2.TDF + 3TC + EFV 3.TDF + 3TC + DTG	48	PC
CR-048	25	Male	5	1.AZT + 3TC + NVP 2.TDF + 3TC + EFV 3.TDF + 3TC + DTG	48	PC
CR-063	47	Female	5	1.AZT + 3TC + NVP 2.AZT + 3TC + EFV 3.TDF + FTC + LPV/r 4.AZT + 3TC + LPV/r 5.ABC + 3TC + LPV/r	Not yet	RF
CR-119	38	Female	14	1.TDF + 3TC + EFV 2.TDF + 3TC + DTG 3.ABC + 3TC + ATV/r	NA	RF
CR-177	40	Male	7	1.AZT + 3TC + NVP 2.TDF + 3TC + EFV 3.TDF + 3TC + DTG	48	PC
CR-226	36	Female	8	TDF + 3TC + DTG	48	PC
CR-335	45	Male	3	TDF + 3TC + DTG	35	PC
CR-339	72	Female	7	1.TDF + 3TC + EFV 2.TDF + 3TC + DTG	47	PC
CR-356	28	Female	2	TDF + 3TC + DTG	47	PC
FD-047	35	Female	8	1.TDF + 3TC + EFV 2.TDF + 3TC + DTG	46	PC
FD-089	38	Female	10	1.TDF + 3TC + EFV 2.TDF + 3TC + DTG	21	PC
FD-096	29	Female	5	1.TDF + 3TC + EFV 2.TDF + 3TC + DTG	21	PC
FD-143	36	Male	10	TDF + 3TC + DTG	16	PC
GD-103	60	Male	6	1.TDF + 3TC + EFV 2.TDF + 3TC + DTG	35	PC
GD-107	43	Female	11	TDF + 3TC + DTG	42	PC
MC-059	64	Male	6	1.AZT + 3TC + NVP 2.TDF + 3TC + EFV 3.TDF + 3TC + DTG	19	PC
MC-111	67	Male	10	1.TDF + 3TC + EFV 2.TDF + 3TC + DTG 3.ABC + 3TC + DTG	23	PC
MC-118	35	Female	8	1. TDF + 3TC + EFV 2. TDF + 3TC + DTG	6	PC
MC-122	59	Female	11	TDF + 3TC + DTG	20	PC
MC-125	19	Male	3	1.TDF + 3TC + EFV 2.TDF + 3TC + DTG	27	PC
MC-210	48	Female	7	TDF + 3TC + DTG	24	PC
MC-222	52	Female	14	1.AZT + 3TC + NVP 2.TDF + 3TC + DTG 3.ABC + 3TC + DTG	38	PC
MC-243	51	Female	10	1.TDF + 3TC + EFV 2.ABC + 3TC + ATV/r 3.ABC + 3TC + DTG 4.TDF + 3TC + DTG	17	PC
MC-339	40	Female	2	1.AZT + 3TC + NVP 2.AZT + 3TC + EFV 3.TDF + 3TC + EFV 4.TDF + FTC + EFV 5.TDF + 3TC + DTG	38	PC
MC-347	18	Female	7	1.AZT + 3TC + NVP 2.TDF + 3TC + DTG	47	PC
MC-354	60	Female	11	1.AZT + 3TC + NVP 2.TDF + 3TC + EFV 3.TDF + 3TC + DTG	47	PC
MC-367	57	Female	10	1.AZT + 3TC + NVP 2.TDF + 3TC + DTG	48	PC
MC-400	40	Female	1	1.TDF + 3TC + DTG 2.ABC + 3TC + LPV/r	NA	RF
MU-050	39	Female	2	1.TDF + 3TC + EFV 2.TDF + 3TC + DTG	22	PC
MZ-030	38	Female	6	1.TDF + 3TC + EFV 2.TDF + 3TC + DTG	18	PC
MZ-113	37	Female	2	1.TDF + 3TC + EFV 2.TDF + 3TC + DTG	35	PC
MZ-207	50	Male	2	1.TDF + 3TC + EFV 2.TDF + 3TC + DTG	32	PC
MZ-262	27	Male	1	TDF + 3TC + DTG	15	PC

*3TC* lamivudine, *ABC* abacavir, *AZT* zidovudine, *DTG* dolutegravir, *EFV* efavirenz, *FTC* emtricitabine, *NVP* nevirapine, *ATV/r* atazanavir boosted by ritonavir, *LPV/r* lopinavir boosted by ritonavir, *PC* policy change (introduction of dolutegravir-based regimen as the first-line treatment in Tanzania), *RF* regimen failure, *NA* not available.

*Regimen were used in a particular order (1, 2, 3, 4 and 5).

Of 33 patients with high viral load (≥ 1000 copies/mL) despite taking ART, 30 (90.91%) were using a DTG-based regimen, mostly TDF + 3TC + DTG, two patients were using ABC + 3TC + LPV/r, and one was taking ABC + 3TC + ATV/r. Of those patients, one had never used a DTG-based regimen but had a history of using TDF and 3TC drugs. Patient MC-243 was already receiving a second line PI-containing regimen, when switching to DTG, suggesting this patient had failed TDF + 3TC + EFV, and might have already accumulated TDF and 3TC resistance. This makes the DTG-containing regimen TLD particularly vulnerable when used after failure of a first line therapy in Tanzania, where first line failure may not have been diagnosed at the time of the switch. The risk of using functional monotherapy is then too high. One patient, CR-063, had not yet been on a dolutegravir-based regimen. Two patients, CR-119 and MC-400, failed their DTG-containing regimen and were already switched to a protease inhibitor-containing therapy when sampled (Table 2).

### HIV drug resistance (HIVDR)-associated mutations

**Figure 2:**
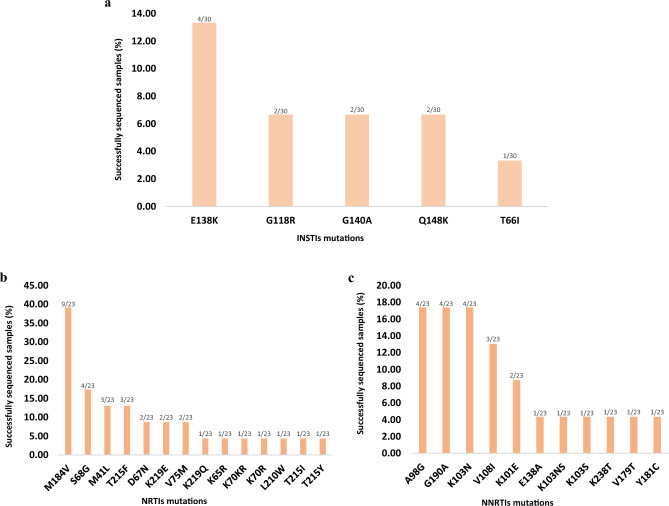
Patterns of HIV drug resistance (HIVDR)-associated mutations. Mutations against integrase strand transfer inhibitors (INSTIs) (**a**). Mutations against nucleoside reverse transcriptase inhibitors (NRTIs) (**b**) and, mutations against non-nucleoside reverse transcriptase inhibitors (NNRTIs) (**c**).

Among NRTI resistance mutations, M184V was the most common 9/23 while for NNRTIs, A98G, G190A, and K103N was the most common 5/23. Furthermore, for INSTIs, E138K was the common 4/30 genotyped mutation (Fig. 2). There were no HIVDR against PIs. The most common HIV-1 subtype was A: 45.16% (14/31), followed by C 35.0% (11/31) then D 9.58% (3/31), and recombinant/ double infection A,D 9.68% (3/31).

### Surveillance drug resistance mutations (SDRMs) and HIV-1 subtypes

Out of 33 samples eligible for genotyping, 93.94% (31/33) samples were successfully genotyped in at least one genomic region, while 66.67% (22/33) were successfully genotyped in all genomic regions tested, protease, reverse transcriptase and integrase (Fig. 1 and Table 3). For one sample, protease and reverse transcriptase were successfully genotyped but integrase failed, and for eight samples integrase was successfully genotyped but protease and reverse transcriptase failed. The reason for this high protease and reverse transcriptase (PRRT) failure rate is not clear, viral load was very high in most of them. In almost half of the genotyped patients (13 of 31), surveillance drug resistance mutations (SDRMs) were detected (Table 3). Five SDRMs (T66I, G118R, E138K, G140A, Q148K) associated with INSTI resistance were detected, of which E138K was the most common 4/30.

**Table 3:** Surveillance drug resistance mutations detected and HIV-1 subtypes for patients with viral loads $\ge$ 1000 copies/mL (N = 33).

Patient ID	Viral load (copies/mL)	Major HIV drug resistance-associated mutations				HIV-1 Sub-type
		NRTI	NNRTI	PI	INSTI	
CR-014	150,000	ND	ND	ND	ND	A
CR-048	2980	D67N, K70R, M184V, T215F, K219E	K103N	ND	G118R, E138K	A
CR-063	6290	M184V	ND	ND	ND	D
CR-119	292,000	M184V	ND	ND	ND	A,D
CR-177	1320	ND	ND	ND	ND	C
CR-226	1210	ND	K103N	ND	ND	C
CR-335	3650	ND	K101E	ND	ND	C
CR-339	1910	GF	GF	GF	ND	C
CR-356	11,100	ND	ND	ND	ND	A
FD-047	36,700	GF	GF	ND	ND	A
FD-089	2840	GF	GF	GF	GF	GF
FD-096	1430	D67N, K70R, M184V, K219E	Y181C, G190A	ND	GF	A
FD-143	103,000	GF	GF	ND	ND	C
GD-103	326,000	ND	K103N	ND	ND	C
GD-107	7540	ND	ND	ND	ND	A
MC-059	12,300	M41L, M184V, T215F	K101E, G190A	ND	ND	A
MC-111	1370	GF	GF	GF	ND	A
MC-118	1500	GF	GF	GF	GF	GF
MC-122	327,000	ND	K103N	ND	ND	A,D
MC-125	284,000	ND	ND	ND	ND	A
MC-210	125,000	GF	GF	ND	ND	A
MC-222	2240	M184V	ND	ND	ND	A,D
MC-243	4000	K65R, M184V, K219Q	K103S, G190A	ND	E138K, G140A, Q148K	A
MC-339	375,000	GF	GF	ND	ND	C
MC-347	58,700	ND	ND	ND	ND	D
MC-354	1390	GF	GF	ND	ND	A
MC-367	13,900	M41L, M184V, L210W, T215Y	G190A	ND	T66I, G118R, E138K	C
MC-400	30,100	ND	ND	ND	ND	C
MU-050	1900	ND	ND	ND	ND	C
MZ-030	128,000	ND	ND	ND	ND	A
MZ-113	3590	GF	GF	ND	ND	D
MZ-207	2130	ND	K103NS	ND	ND	A
MZ-262	2830	ND	ND	ND	ND	C

*NRTI* nucleoside reverse transcriptase inhibitors, *NNRTI* non-nucleoside reverse transcriptase inhibitors, *PI* protein inhibitor, *INSTI* integrase strand transfer inhibitor, *ND* none detected, *GF* genotyping failure.

### Prevalence of surveillance drug resistance mutations (SDRMs)

In total 23 sequences were analyzed for protease and reverse transcriptase (PRRT) mutations (Table 4). Of which 13 sequences contained RT SDRMs (56.5%). Thirty sequences were analyzed for integrase where 3 sequences (10.08%) contained INSTI SDRMs. Worrying is that for those samples where full genotypic information was available (n = 22), all three with INSTI SDRMs also had NRTI and NNRTI SDRMs, adding up to almost 14%.

**Table 4:** Detected surveillance drug-resistance mutations.

Resistance category	No. analyzed	No. containing SDRM	Percentage (%)
Sequences with any SDRM	31	13	41.39
PR Sequences with PI SDRMs	23	0	0.00
RT Sequences with NRTI SDRMs	23	8	34.88
RT Sequences with NNRTI SDRMs	23	10	43.50
RT Sequences with NRTI and NNRTI SDRMs	23	5	21.70
IN Sequences with INSTI SDRMs	30	3	10.08
Sequences with NRTI, NNRTI, and INSTI SDRMs	22	3	13.63

*IN* integrase, *INSTI* integrase strand transfer inhibitor, *NNRTI* non-nucleoside reverse transcriptase inhibitor, *NRTI* nucleoside reverse transcriptase inhibitor, *PI* protein inhibitor, *PR* protease, *RT* reverse transcriptase, *SDRM* surveillance drug resistance mutation.

### Drug susceptibility assessment

**Figure 3:**
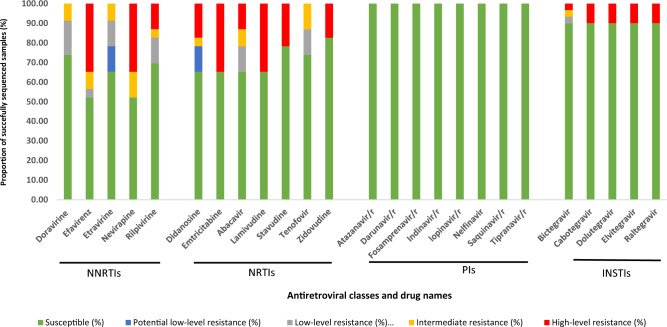
Antiretroviral drugs resistance and susceptibility profile among the successfully genotyped patients samples whose viral loads were $\ge$ 1000 copies/mL.

The drug susceptibility assessment based on the patterns of mutations per patient is illustrated in Fig. 3. The detection of high-level resistance against NRTs and NNRTIs was consistent with patients' treatment history. Furthermore, patients with INSTI mutations and high level INSTI resistance (MC-243, MC-367 and CR-048) had a history of using DTG-based regimen (Table 2).

To assess whether the estimated susceptibility was relevant for the concurrent treatment regimen, a GSS to the concurrant regimen was calculated for the 22 for whom the full PRRT and INSTI genotype was available (Table 5). This revealed that 68.18% (16/22) had a total GSS of $\ge$ 2.5 towards the concurrent regimen, suggesting that for the majority of patients other factors than their drug resistance profile was causing unsuppressed viral load. For one patient, the total GSS was 2.0, but with an active DTG. Careful management and consideration of alternative therapeutic options may be necessary for this patient to maintain effective treatment and prevent further resistance development especially to DTG. Three had a total GSS less than 1, and two had a total GSS of 1.5. All three patients with INSTI resistance mutations were highly resistant to DTG, the other patients are at risk for developing DTG resistance (Table 5). This highlights the importance of monitoring and potentially adjusting treatment to avoid virologic failure and further increase of drug resistance in these individuals. Given that time since HIV diagnosis and whether or not there were other chronic diseases was associated with unsuppressed viral load (Table 1), we added this information to the table for illustration purposes.

**Table 5:**
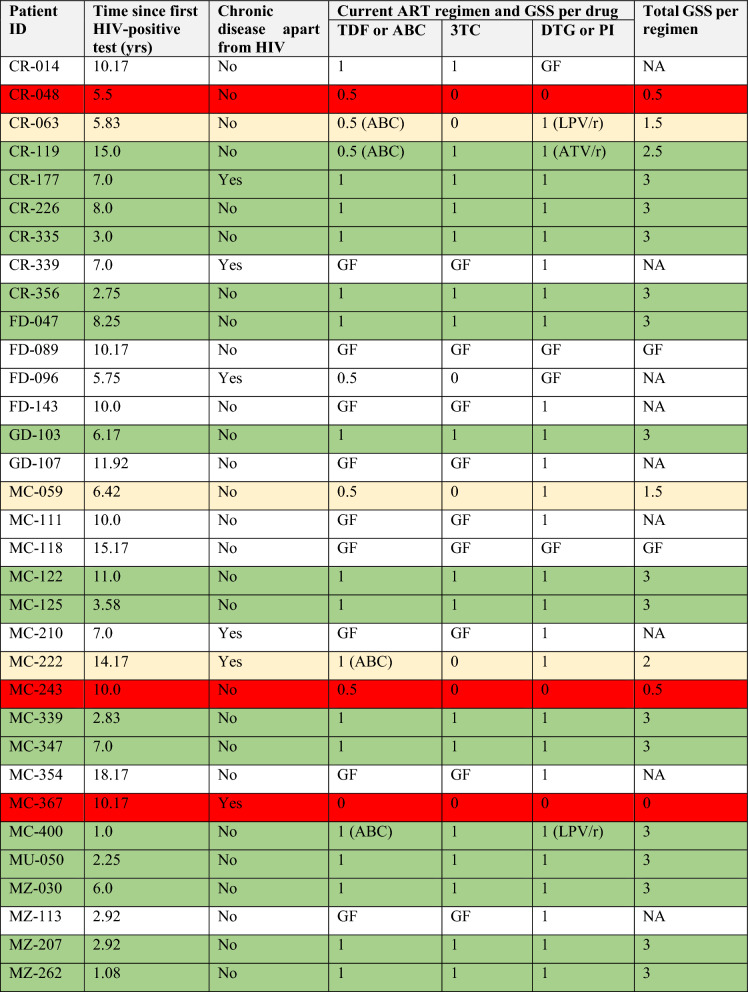
Genetic susceptibility score (GSS) to the concurrent regimen for patients with viral loads $\ge$ 1000 copies/mL (N = 33).

The regimen for all patients was TDF + 3TC + DTG, except for CR-063 and MC-400 it was ABC + 3TC + LPV/r, CR-119 had ABC + 3TC + ATV/r, MC-111 and MC-222 had ABC + 3TC + DTG.

*TDF* tenofovir, *ABC* abacavir, *3TC* lamivudine, *DTG* dolutegravir, *ATV/r* atazanavir boosted by ritonavir, *LPV/r* lopinavir boosted by ritonavir, *GSS* genetic susceptibility score, *GF* genotypic failure, *NA* not applicable because of some missing value(s).

## Discussion

This study aimed at determining the prevalence of viral suppression and HIV drug resistance (HIVDR) among a random sample of 600 HIV treatment-experienced adults (18 years and above) living in Ukonga and Gongolamboto, Dar es Salaam, three years after the introduction of dolutegravir (DTG)-based antiretroviral therapy (ART). The survey constitutes a baseline at the study site, in order to inform and evaluate upcoming intervention studies. In this survey the prevalence of viral suppression (defined by viral load (VL) < 1000 copies/ml) was 94.48%, this is a substantial milestone on the last Joints United Nations AIDS Programme on HIV/AIDS^3^ (UNAIDS target where 95% of people on ART should have suppressed their viral loads by 2025).

The high virological suppression rate in the studied area is probably linked to the policy change of replacing non-nucleoside reverse transcriptase (NNRTI)-based regimen by DTG-based regimen (tenofovir + lamivudine + DTG or TLD) in first line. This policy change resulted in 99% of the surveyed patients taking the TLD regimen. The treatment success rate in our survey is consistent with the recent national representative survey carried out in 2021, in which viral suppression among adults was 96.16%^15^. The national survey was conducted 18 months after DTG introduction, while patients in our survey were on average already three years on a DTG-containing regimen. Prior to DTG, viral suppression rate was 87.7% among patients with age between 15 and 64 years, as documented in the Tanzania national survey of 2016–2017^41^, the last survey before introduction of DTG-based regimen in 2019^7^. This shows that the introduction of DTG-based regimen successfully managed to bring treatment success to the majority of treated patients. Previously, Kiekens et al.^24^ described that on a broader scale, the accessibility of highly potent ART combined with second-generation integrase inhibitors, which possess a robust genetic resistance barrier, presents a novel and encouraging avenue for treatment^24^. The dependence on new ART options should be addressed, especially considering that there are currently limited new ART medications in development. The focus should be on optimizing the use of existing ART regimens and exploring alternative strategies to manage HIV and prevent HIVDR, rather than anticipating the introduction of new drugs in the near future. As Kiekens et al. posit, overreliance on new drugs is one of the drivers of HIVDR.

While treatment success was high, the proportion of those harbouring any SDRMs among those with unsuppressed viral load (VL ≥ 1000 copies/mL) was also high (41.39%). Treatment success viral load thresholds are much higher in Tanzania^2^ compared to Western countries^44,45^ (which aim for VL < 50 instead of 1000 copies/mL). In addition, Tanzanian guidelines allow HIVDR testing^2^ only for samples with unsuppressed viral load. This study adhered to Tanzanian guidelines to ensure that the study followed local protocols, as the current survey is intended to be repeated in the context of local interventions consistent with the healthcare practices and infrastructure in the region. The high proportion of SDRM is therefore not surprising. Resistance was most commonly observed against NNRTIs (52.1%) and NRTIs (34.88%) similar as in other studies in Tanzanian^15,16,42,43^. The analysis of PRRT mutations in 23 sequences revealed SDRMs in 56.5% of the studied patients, while no protease SDRMs were detected. This is consistent with the history of treatment guidelines in Tanzania. In the period 2006^5^–2018, the first treatment line included 2NRTIs plus one NNRTI. PIs were, and still are, mainly used for patients who failed the first line regimen^7^. Comparatively, the examination of integrase sequences from 30 samples revealed that 10% contained INSTI SDRMs. The detected INSTI mutations, T66I, G118R, E138K, G140A, and Q148K are associated with reduced susceptibility to DTG. E138K and G140A also affect elvitegravir and cabotegravir, in combination with Q148A, G140A reduces the susceptibility to raltegravir and elvitegravir by more than 100-fold while susceptibility to dolutegravir and bictegravir could be reduced by up to fivefold. T66I mainly affects the susceptibility of EVG by tenfold, with a minimal effect to other INSTIs^33^.

The proportion with INSTI mutations is higher than the previously documented prevalence of 5.8% by Kamori et al^15^. This indicates a potential increase in the occurrence of INSTI resistance mutations, which could have significant implications for the effectiveness of integrase-targeting antiretroviral therapies. However, it is important to consider the scope and context of both studies when interpreting and comparing these findings. We found that HIV-1 subtype was A: 45.16% (14/31), followed by C 35.0% (11/31) then D 9.58% (3/31). Our results agree with the findings from the neighbouring country Kenya which reported subtype A (70.3%) as the dominant subtypes^46,47^. The Tanzanian study revealed subtype C (60.87%), followed by subtype A (41.30) were the commonly prevailing subtypes among adolescents and young adults^48^. A national representative survey found that C was the predominant circulating HIV-1 subtype in adults, 45.3% followed by A, 35.7%^15^. Globally, subtype C accounts for 48% of worldwide HIV infections, primarily clustered in eastern and southern Africa, while subtype A (12%) and subtype D (2%) constitute subsequent proportions^49^.

Our data substantiate the ongoing concern regarding the development of drug resistance mutations in reverse transcriptase, which can compromise the efficacy of antiretroviral therapies given that nucleoside reverse transcriptase inhibitors (NRTIs) are the backbone of any regimen, including^8,42,43,50^. The most worrying is that in our study, 13.63% of successfully sequenced patients’ samples for PRRT and integrase had triple class resistance; NRTI and NNRTI and INSTI. Given that patients had been switched to DTG because of the policy change and not because of treatment failure, the high levels of NRTI and NNRTI resistance would indicate that these patients may not have been followed up closely enough to timely switch therapy. This may have contributed to the surprisingly high rate of unsuppressed viral load under the DTG-regimen, some with but most without DTG resistance, after only three years of DTG-containing therapy. This was anticipated in our previous research when considering the drivers of HIVDR at systems level^24^.

Unique in our study is that we had treatment information, this helped to assess the relevance of the resistance profile for the therapy that the patients were receiving, we examined their GSS to the concurrent regimen. Surprisingly, 68.18% of patients with unsuppressed viral load exhibited GSS values of ≥ 2.5 for their concurrent regimen, implying that factors other than their drug resistance profiles might be contributing to the unsuppressed viral load. This underscores the complex interplay of multiple factors beyond resistance mutations, including issues related to adherence, healthcare access, and psychosocial aspects^24^. This multifaceted perspective on treatment failure emphasizes the need for a holistic approach to understanding and managing HIV treatment outcomes^25^. Those with a low GSS but an active DTG face the risk of developing DTG resistance. This underscores the significance of continuous monitoring and timely treatment adjustments to prevent virologic failure and subsequent resistance escalation^13^. In addition, knowledge about drug resistance development and treatment adherence^24,25^ are also important. The patients with DTG resistance also exhibited resistance to reverse transcriptase (RT) inhibitors. We may speculate here that DTG resistance mainly developed in patients that had already failed their first line regimen with reverse transcriptase inhibitors, a failure that might have gone undiagnosed. These patients might have accumulated DTG resistance because they had in fact been receiving functional monotherapy. In countries that switch all first line patients to TLD, this DTG-containing regimen may be at risk when first line failure went undiagnosed.

Despite its effectiveness, the emergence of dolutegravir resistance poses a significant concern in HIV treatment^15,16^. In high-income countries with well-established healthcare systems and access to a diverse range of antiretroviral drugs, the prevalence of dolutegravir resistance remains relatively low even years after its introduction (below 3%)^51^. This can be attributed to regular treatment monitoring, early detection of resistance, and the availability of alternative treatment options, which collectively contribute to a more controlled resistance landscape. However, in low and middle-income countries, where healthcare resources and treatment options may be limited, the prevalence of dolutegravir resistance becomes more concerning ranging from 1 to 20%^52^. Factors such as delayed diagnosis, restricted access to resistance testing, and suboptimal adherence to treatment can facilitate the development and dissemination of resistance mutations in these settings^17^. This shows how development of HIVDR is context dependent, and battling HIVDR has to be done at a systems level.

In the regression analysis, we found that patients diagnosed since less than 2 years had lower odds (0.13) of having an unsuppressed viral load as compared to those with at least 10 years since diagnosis. This could be attributed to various factors, such as closer monitoring, more accessible healthcare services, and timely interventions during the early stages of diagnosis for patients that were diagnosed under the test and treat strategy^7^. This finding aligns with expectations in resource-constrained settings, where recently diagnosed individuals might receive more focused attention and support (unpublished data). Surprisingly, patients self-reporting other underlaying chronic disease conditions also had lower odds (0.18) of unsuppressed viral load as compared to their counterparts. This finding suggests that patients with coexisting chronic diseases might be receiving more comprehensive medical care and support, which inadvertently contributes to better HIV treatment outcomes. This observation underscores again that HIVDR has to be addressed at systems level.

While interpreting the results of our study, it is crucial to acknowledge certain limitations that might affect the generalizability of the findings. Firstly, our research was limited to the urban patient, potentially excluding valuable insights from rural or suburban areas, where healthcare access and disease patterns may differ. Secondly, we excluded patients who were not living in the study area, while attending clinics in the study area. This points to a subset of patients (60%) who attend clinics away from the area they live in, also for our study area, we are aware of patients attending clinics in other areas, mostly to avoid stigma. The absence of these patients in our survey may affect the results. It is not clear whether this may underestimate (those experiencing or fearing more stigma are absent in our survey) or overestimate (attending clinics away from where you live may reduce the consequences of stigma) treatment success in our survey.

Our study focused on patients who were on treatment for at least one year (13% had less than 1 year of ART in our study area), which might overlook potential differences in disease progression and treatment response among those with shorter treatment durations. Additionally, by only considering participants aged 18 years and above, we may have missed important aspects of the condition's impact on younger age groups, for whom treatment approaches and outcomes could vary significantly. Given the low success rate of PRRT genotyping, caution should be exercised when interpreting the genotyping results and drawing conclusions based solely on the genotyped data. Our interpretation, mostly considered the samples successfully genotyped for PPRT and integrase. Another limitation was that our dataset included information on the previous treatment regimen only for patients with unsuppressed viral loads. In addition, as per Tanzanian guidelines, we were not able to assess the presence of HIVDR mutations in patients with a detectable viral load below 1000 copies/mL. As a result, we were unable to ascertain the confounding effect of potential pre-existing HIVDR mutations in patients who transitioned to DTG-containing regimen from a different first line regimen. Whether and how much this could have influenced the observation that patients diagnosed since less than 2 years had lower odds of unsuppressed viral load can therefore not be estimated.

## Conclusions

This study represents a foundational baseline investigation conducted in a designated intervention site, providing comprehensive insights into viral suppression, HIVDR and assessing GSS as a multifaceted metric to evaluate treatment effectiveness. In our study site, approximately 95% of patients achieved viral suppression under therapy, successfully reaching the last of the 95–95–95 UNAIDS targets. This achievement was attributed to the replacement of NNRTIs with DTG in the first-line treatment, which played a significant role. However, it is essential to note that HIVDR mutations, including INSTIs resistance mutations, were detected in this survey. Given that the overall viral suppression rate is high, the prevalence of dolutegravir resistance remains low but concerning, reaching already 10% of genotyped patient, all with triple class resistance. Without appropriate interventions, HIVDR could potentially threaten the effectiveness of the currently available treatment regimen with INSTIs. Therefore, it is crucial to maintain high patient levels of viral suppression and continually survey for HIVDR to integrase inhibitors. The fact that in most of those with unsupressed viral load, the estimated activity of the treatment was still sufficient to suppress viral replication, shows how important other factors are for treatment success, and that a systems approach is needed.

We strongly recommend the design and implementation of high-impact interventions to prevent any further increase in HIVDR in both the study area and Tanzania as a whole. This action is vital to safeguard the potency and effectiveness of this new class of INSTIs. To ensure successful interventions, a transdisciplinary human-centered approach should be adopted, examining the problem at a systems level. A systems approach offers a holistic vision on the complex issue of HIVDR, recognizing the interconnected nature of medical, social, and systemic factors that contribute to treatment effectiveness. This comprehensive strategy will help address the challenges associated with HIVDR and secure the long-term success of treatment efforts.

## Data Availability

Data used for analysis to produce this manuscript are available for secondary analysis from the corresponding author or the DUCS Platform database management upon request.

## Abbreviations

ART: Antiretroviral therapy
CTC: Care and treatment clinic
DUCS: Dar es Salaam urban cohort study
DTG: Dolutegravir
HIVDR: HIV drug resistance
NRTIs: Nucleoside reverse transcriptase inhibitors
NNRTIs: Non-nucleoside reverse transcriptase inhibitors
PIs: Protein inhibitors
INSTIs: Integrase strand transfer inhibitors
ARVs: Antiretrovirals
TLD: Tenofovir plus lamivudine plus dolutegravir
PRRT: Protease and reverse transcriptase
